# A trans-scalar approach to peacebuilding and transitional justice: Insights from the Democratic Republic of Congo

**DOI:** 10.1177/00108367211059448

**Published:** 2021-12-29

**Authors:** Sara Hellmüller

**Keywords:** DR Congo, peace research, peacebuilding, transitional justice, trans-scalarity

## Abstract

Peace research has taken a local turn. Yet, conceptual ambiguities, risks of romanticization, and critiques of co-option of the “local” point to the need to look for novel ways to think about the interactions of actors ranging from the global to the local level. Gearoid Millar proposes a trans-scalar approach to peace based on a “consistency of purpose” and a “parity of esteem” for actors across scales. This article analyzes the concept of trans-scalarity in the peace process in Ituri, a province in the northeastern Democratic Republic of Congo (DRC). Drawing on qualitative data from more than a year of research in the DRC, I argue that while a trans-scalar approach was taken to end violence, it was not applied to transitional justice initiatives. The result was a negative, rather than a positive peace. By showing the high, but still untapped, potential of trans-scalarity, the article makes three contributions. First, it advances the debate on the local turn by adding empirical insights on trans-scalarity and further developing the concept’s theoretical foundations. Second, it provides novel empirical insights on the transitional justice process in the DRC. Third, it links scholarship on peacebuilding and transitional justice, which have often remained disconnected.

## Introduction

Peace research has taken a local turn. In a wave of criticism of the liberal approach to peacebuilding, a consensus emerged among peace scholars that increased attention to local actors, capacities, and perceptions was indispensable to build lasting peace (Autesserre, 2010; Mac Ginty and Richmond, 2013; Paris, 2002). Scholars emphasized the need to re-conceptualize the “local” in the strategy, implementation, and outcomes of peacebuilding (Campbell et al., 2012: 4; Tadjbakhsh, 2011: 4). However, besides ambiguity about what the “local” means (Buckley-Zistel, 2021; Kappler, 2015) and the potential romanticization of everything “local,” critiques have also been raised about the co-option of the local turn by liberal peacebuilding (Richmond, 2012). Rather than leading to an emancipatory version of peacebuilding based on genuine cooperation between actors across different levels, the turn toward the local was said to deepen the imposition of liberal values because it came to mean that local actors should own externally designed projects (Hellmüller, 2012; Von Billerbeck, 2016). In an attempt to overcome these challenges and go beyond binary conceptions of “local” versus “international” actors, critical peace scholars suggested to focus on hybridity (Mac Ginty, 2010; Richmond and Mitchell, 2012; Tom, 2013) or friction (Björkdahl et al., 2014; Björkdahl and Höglund, 2013) in peacebuilding. Yet, as Millar (2020: 274) argues, in order to counterbalance the dominance of the international, scholars often overemphasized local agency and neglected the influence of the broader global context in which conflicts are located. As an alternative, he suggests a “trans-scalar peace system” based on a “consistency of purpose” and a “parity of esteem” for actors across global, regional, international, national, and local scales (Millar, 2021).

Research on transitional justice has also taken a local turn. Similar to peace scholars, authors studying transitional justice point to the inadequacy of international template approaches (Gready and Robins, 2014; Iliff, 2012; Shaw et al., 2010) and recognize that a more nuanced understanding of local transitional justice is needed (Kochanski, 2020). However, while it is now widely acknowledged that transitional justice needs to be firmly grounded in local contexts, the way in which the actors involved in transitional justice processes interact across different levels has not been sufficiently explored.

This article fills this gap by analyzing the concept of trans-scalarity proposed by Millar (2021) in the peace process in Ituri, a province in the northeastern Democratic Republic of Congo (DRC), from 1999 to 2003. I argue that while the efforts to end physical violence in Ituri were trans-scalar, this was not the case for transitional justice initiatives. The overall result was a negative peace, defined as an end of active and widespread violence, and not a positive peace, which would have included a comprehensive transformation of relationships (Galtung, 1969).

The article is based on empirical data gathered between 2011 and 2014 through over 130 interviews, focus group discussions (FGDs), and informal discussions with more than 195 persons in the DRC. It is structured into two parts. In the theoretical part, I review the literature on the local turn in transitional justice and show how a trans-scalar approach could help move the debate on local-international interactions forward. In the empirical part, I analyze trans-scalarity in the peace process in Ituri by comparing the approaches taken to end violence with those taken to promote transitional justice.

By showing the high, yet often untapped, potential of a trans-scalar approach, the article makes three contributions. First, it provides a nuanced analysis of the local turn in the peacebuilding and transitional justice literature by adding empirical insights on trans-scalarity and by further developing its theoretical foundations. Second, the article extends the existing body of knowledge on transitional justice by providing novel empirical insights from the DRC, a relatively understudied context in the transitional justice literature.^1^ Finally, the article links scholarship on peacebuilding and transitional justice, thereby combining two sub-fields of peace research that are closely related, but have often remained disconnected (Millar and Lecy, 2016; Sharp, 2013).^2^

## Theoretical framework

### Local turn in transitional justice

Research and practice on transitional justice have taken a local turn (Kochanski, 2020; Shaw et al., 2010). As part of a broader critical turn in the literature (Sharp, 2019: 576–580), the importance of acknowledging local capacities, institutions, and processes has been firmly acknowledged (Huyse and Salter, 2008; Iliff, 2012; Quinn, 2007). Many authors see the local turn as a hopeful alternative to the dominant script of transitional justice that has mostly taken a top-down approach and emphasized atrocity justice (Boege and Rinck, 2019; Ferati-Sachsenmaier, 2019; Sharp, 2018; Verovšek, 2019: 704). They point to the risks involved if international transitional justice processes are distant from local concerns or if they hijack local efforts at societal reconciliation, thereby further challenging the already fragile situations in many conflict contexts (Ferati-Sachsenmaier, 2019; Verovšek, 2019). They suggest to return the “local” to its rightful place—that is, recognizing it as a crucial environment where multiple actors and networks exist, interact, and overlay. This is often accompanied by a call for legal pluralism that acknowledges the different legal systems in a society based on a broad conception of justice (Kochanski, 2020: 29; Merry, 2006: 870; Quinn, 2007).

While not generally opposed to giving local actors a stronger role, other authors tell a cautionary tale about the benefits of local transitional justice. They criticize their scholarly counterparts for sustaining a normative bias by idealizing and romanticizing the “locals” and for relying on weak empirical data and evidence to support their claims of effectiveness (see Kochanski, 2020; Piccolino, 2019). They point out that traditional legal systems are ill-prepared to tackle serious offenses of mass crimes and express their concerns about due process (Meyerstein, 2007). They also denounce the sometimes discriminatory settings of traditional institutions, such as the gender biases of certain indigenous rituals and the ageist hierarchies that in some cases contributed to the onset of war (Branch, 2014; Friedman, 2015). Moreover, they point to the fact that local justice systems are vulnerable to being distorted by interests of the national government and political elites (Brown and Sriram, 2012; Sharp, 2019; Snyder and Vinjamuri, 2006; Subotić, 2009), a factor that is often overlooked in local transitional justice accounts (Piccolino, 2019: 358).

Despite some discrepancies of opinion on how central the local should be in transitional justice and on the efficiency of local processes, authors seem to agree that international transitional justice efforts cannot be fully successful if they do not connect with local communities. They posit that the international and the local are two permeable and interconnected entities (Sharp, 2018). This is also the state of consensus in peace research more generally (Björkdahl et al., 2014; Randazzo and Torrent, 2021) and several authors underline the need to analyze transitional justice in the framework of broader peacebuilding strategies (Sharp, 2018; Sriram, 2007, 2009a). In this article, I provide such an analysis of the interactions of actors across local and international levels drawing on the concept of trans-scalarity as developed by Millar (2020).

### Trans-scalarity

Millar (2021: 646) points to the “complex global dynamics” of conflicts and their multi-layered nature and thus calls for a “trans-scalar peace system,” which includes actors across the global, regional, international, national, and local levels. He discusses two aspects of such an approach. First, he calls for a “consistency of purpose” (Millar, 2021: 646). This means that policies and actions become “coherent and supplementary” across scales as they are guided by a “collective and coherent vision for peace” (Millar, 2021: 641, 649). Second, he underlines the importance of “a parity of esteem” (Millar, 2021: 648) for actors on all scales while privileging “the voice of those with the most pertinent knowledge, experience and capacity for action” (Millar, 2021: 640). In other words, he calls for a division of tasks according to each actor’s comparative advantage so that those actors closest to where the implementation of any given program occurs are privileged in designing the policy guiding that action (Millar, 2021: 648).

While providing a novel conceptual framework, the trans-scalar approach can be further developed theoretically in at least two ways. First, Millar (2021) explores trans-scalarity in the context of a positive peace system. He argues that structural violence at the global level tends to produce situations of negative peace while only a trans-scalar peace system can achieve a positive peace (Millar, 2021: 646). While appreciating his attempt to think about a comprehensive global peace system, I argue that to further develop our thinking on trans-scalarity, we need to both *extend* and *disaggregate* the analysis. On the one hand, trans-scalarity may not only be needed for positive peace, but also for negative peace. We should thus *extend* the analysis to negative peace to examine the potential of trans-scalarity across a wider spectrum of purposes. On the other hand, positive peace consists of many different aspects, related for instance to security issues, the political framework, socio-economic foundations, or reconciliation and justice in a given context (Smith, 2004). To produce finer-grained insights, we should therefore *disaggregate* our analysis to examine trans-scalarity with regard to the specific components of a positive peace. Based on these two considerations, I analyze the potential of a trans-scalar approach both in terms of ending physical violence and establishing a negative peace as well as in terms of transitional justice initiatives as one component of a positive peace.^3^

Second, Millar (2020: 270) conceptualizes peacebuilding as being “characterized as much by the unintentional, the uncontrollable, and the unpredictable as it is by intention and agency” and hopes for a development from an “initially intentional convergence across scales” into “a more habitual coherence” (Millar, 2021: 650). In order to operationalize trans-scalarity, it is relevant to further unpack this distinction between unintended and intended convergence of purpose and to explore situations where trans-scalarity came about unintentionally as well as situations where it needed intentional coordination. Such an analysis, which I provide in the following, uncovers the enabling conditions for trans-scalarity and generates insights on how it can be promoted.

## Trans-scalarity in Ituri

### Methodological note

To analyze trans-scalarity in Ituri in the DRC, I draw on empirical data collected in five research stays of more than a year in total between 2011 and 2014.^4^ I spent most of the time in Ituri and collected data in 21 different villages,^5^ including the provincial capital town of Bunia, but I also went twice to the capital city Kinshasa where some international organizations and most national elite actors are located.

I conducted over 130 interviews, FGDs, and informal discussions (see Online Appendix) with more than 195 persons, and did participant observation. The method I used most often was in-depth semi-structured interviews. The main respondents were local and international peacebuilders at the center of a trans-scalar approach. To provide a thick analysis of the context and outcomes of peacebuilding efforts, I also interviewed local population groups,^6^ including local chiefs, local and national government actors, former belligerents, professors, and external experts. I complemented these interviews with FGDs to gather the perspectives of different population groups.^7^ I used FGDs in order not to take too much time from respondents who had to take care of their cattle or fields, but also to engage them in a discussion enabling me to observe points of consensus and disagreements. Moreover, I held countless informal discussions to gather information, clarify issues, probe hypotheses, and fill data gaps.^8^ I also did participant observation, joining local and international organizations on strategic retreats, on field trips to visit their projects, and in their daily work at their offices in Bunia.

I identified respondents through theoretical sampling, meaning to approach those persons who are likely to “provide the most information-rich source of data” (Birks and Mills, 2011: 11). This meant that I selected interviewees based on their insights on the conflict and the peace process as well as their roles in it, so as to ensure a variety of perspectives. I complemented this method with snowball sampling, asking respondents to indicate further potentially relevant persons. I documented all conversations and observations by taking notes and recorded the conversations if interviewees agreed. To protect the respondents’ identity, all interviews were conducted in a confidential manner. For data collection in rural villages, I had interpreters. I transcribed all interviews and FGDs in French and translated cited passages into English. I identified the point of saturation of data collection when additional data no longer revealed new insights, but only confirmed existing ones that were already sufficiently validated (Birks and Mills, 2011: 10). I analyzed data with MaxQDA software according to the constant comparison method (Glaser and Strauss, 1967: vii; Strauss and Corbin, 1994: 273). This means that I structured the data according to different codes and categories and then compared codes to codes, codes to categories, and categories to categories while at the same time gathering new data and comparing it to the existing codes and categories (Birks and Mills, 2011: 11). Thereby, I iteratively built the argument made in this article (Yin, 2009: 141–144).

Throughout the research process, I used three ways to minimize bias (Wood, 2007). First, I triangulated data to cross-validate findings (Baxter and Jack, 2008: 556; Thies, 2002: 357). Second, I constantly questioned my research for biases. I re-read interview or FGD questions for their potential suggestive character and tried to phrase them as openly as possible (except for questions of clarifications). I analyzed interviews and FGDs for potential biases immediately after I had conducted them, as well as during the transcribing, coding, and write-up process. Third, I carefully analyzed metadata, defined by Fujii (2010) as “informants’ spoken and unspoken thoughts and feelings which they do not always articulate in their stories or interview responses” (p. 231). After an interview or FGD, I noted my thoughts about the conversation, which increased my awareness of potential biases.

### The complex conflict system in Ituri

Millar’s suggestion for a trans-scalar peace system stems from the acknowledgment that conflicts are complex global systems that play out across different scales (Millar, 2020, 2021). The conflict in Ituri illustrates such a complex conflict system across global, international, regional, national, and local scales (Veit, 2010; Vircoulon, 2005).^9^

At the national level, shortly after the end of the so-called first Congo War (1996–1997), which terminated the 32-year dictatorship of Mobuto Sese Seko, the Second Congo War broke out in 1998 between then President Laurent-Désiré Kabila and different rebel groups that were backed by Rwanda and Uganda. The parties signed a peace agreement on 2 April 2003, which contained the so-called “Global and Inclusive Agreement” foreseeing a democratic transition that ended with elections in 2006 (Autesserre, 2010: 51). At the local level in Ituri, the violence opposed the two main ethnic groups, Hema and Lendu. It erupted around a land conflict in an area called Walendu Pitsi in 1999 (Byensi Mateso, 2009: 9). The dispute had started in April of the same year between a landowner, Singa Kodjo, and neighboring communities.^10^ Kodjo was a member of the Hema ethnic group and the communities mainly came from the Lendu ethnic group. Kodjo alleged that he had acquired the land around his property officially, but Lendu farmers who lived on the land had not been informed and refused to vacate it (Byensi Mateso, 2009: 9). Such land evictions, mostly by Hema landowners at the expense of Lendu farmers, had become quite common. The dispute thus quickly escalated into violence, which spread throughout Ituri. The embroilment with the Second Congo War ongoing at the national level led to the formation of heavily armed local militias and to large-scale massacres (Vircoulon, 2010: 209; Vlassenroot and Raeymaekers, 2004: 391; Wolters, 2005: 2).^11^

The following three main issues characterized the conflict in Ituri: *Ethnicity*, *land*, and *governance*, and they were all trans-scalar (Hellmüller, 2018). First, aspects across scales influenced *ethnicity* as a local conflict factor. The Belgian colonial administration (1908–1960) had created a distinct elite composed mostly of Hemas, which fueled conflicts between Hemas and Lendus and installed in them a sense that they were incapable of cohabiting peacefully (Kaputo, 1982; Pottier, 2008; Prunier, 2008). Mobutu further built on this conflictual relationship through his policy of “divide and rule.” Moreover, when the Second Congo War broke out, several rebel groups manipulated ethnic alignments to recruit local fighters (Hellmüller, 2019; Veit, 2010).

Second, aspects across scales also influenced *land* as a conflict issue. National land legislations during colonial times and under Mobutu created insecurity of land ownership for Lendu communities. Thus, land evictions, such as the one by Kodjo that happened in 1999, had the potential to escalate quickly (Lobho, 2002: 75; Van Woudenberg, 2004: 192). Moreover, access to land meant access to strategic roads and border crossings, which attracted the interest of national rebel leaders during the Second Congo War as well as regional actors, such as Uganda, who further fueled the conflict (Fahey, 2009). More broadly, the DRC is rich in natural resources and access to its lands is thus a high-stakes issue of global magnitude (Carayannis, 2009).

Finally, aspects across scales also influenced *governance* mechanisms. Local chiefs could not prevent the conflict from escalating into violence since they were often themselves involved in it (Maindo Monga, 2003: 183; Muhigi Barozi, 2010: 71). The Second Congo War ongoing at the national level further accentuated the governance crisis. While some rebel leaders controlling Ituri played a conciliatory role as ethnic violence threatened their capacity to govern, their turnover was so high that they did not have a lasting impact (RHA IKV and Pax Christi, 2013). Moreover, Ugandan soldiers, who were stationed in Ituri, mostly sided with Hema landowners who had become important trade partners for Uganda (Prunier, 2008: 43).^12^

This account of the armed conflict underlines its complexity and the concomitant influence of global, international, regional, national, and local aspects on the three main conflict issues. To promote peace in such a multi-layered system, a trans-scalar response is needed. Yet, as shown in the following, while trans-scalarity was present to end violence, this was not the case for longer-term peacebuilding efforts, such as transitional justice initiatives. The result was a negative, rather than a positive peace.

### Trans-scalarity in ending violence

Widespread inter-ethnic warfare in Ituri ended in 2003.^13^ Fatality numbers related to the inter-ethnic violence decreased from 1’946 in 2002 to 258 in 2003 to below 25 in 2004 (Sundberg et al., 2012).^14^ Iturians experienced this end of widespread violence as a profoundly local process. Anecdotal evidence from interviews suggests that a shift in mind-set happened in the population. According to the local narrative, they realized that national elite actors had manipulated them, leading to enormous human and economic damage. As one interviewee stated, “on both sides, we realized that we had lost—material losses, but also human losses.”^15^ Consequently, they launched efforts to end the violence. In several villages, one ethnic group invited the other to share meals or drinks again. They sent either a representative or a letter to inform the other community of their intention to end the fighting. One interviewee said,

One day, the Lendus decided to bring some food—bananas, manioc, flour—all the products that are not available here because we do not have these fields. When the people here saw it, they were afraid that this was just another way of [. . .] poisoning them. They went to see their local chief. The Lendus said, “we want to bring you food because you cannot only eat fish, you must be hungry, so we brought you something else.” The local chief [. . .] explained that from now on, the Lendus were their brothers again, that they had been manipulated by politicians, but that they should remember that they had lived side by side for a long time.^16^

Local peacebuilders supported these processes. As most of them were associated with either the Hema or the Lendu ethnic group according to the composition of their staff, they had to join forces if they wanted to remain active in the ethnically divided environment. One of the most telling examples is the *Réseau Haki na Amani*, a network of different local organizations that formed during the war (Mongo and van Puijenbroek, 2009: 7). As one of the founding members said, “it was obvious for everybody that it was impossible to work by ourselves.”^17^ By working together, these networks gave an important example of inter-ethnic cooperation amid ongoing tensions. Moreover, local organizations also engaged in vast awareness-raising activities to spread messages of peace. They organized peace caravans and days of peace and conducted radio programs and workshops with local chiefs, militia members, religious actors, women, and youth. The *Associations des Mamans Anti-Bwaki* (AMAB), for instance, initiated dialogues between women of different ethnic backgrounds. These women then raised awareness for the need to end violence in their families and communities and formed so-called “peace chains” with women leaders who spread messages of peace at the village level.

On their side, international actors also wanted to end the violence at all costs. This was of particular urgency after the nation-wide peace agreement was signed on 2 April 2003, as the atrocious massacres in Ituri threatened the foreseen political transition. Therefore, international actors supported a separate peace process for Ituri. The UN presided over a Pacification Commission that brought together 177 delegates composed of the conflict parties in Ituri, government representatives from the DRC and Uganda, as well as civil society actors. The talks ended on 14 April 2003 with the decision to create a Special Interim Administration for Ituri (Boshoff, 2003). Moreover, in a record-breaking time, the UN authorized *Artemis*, a military operation under French command with more than 1200 troops, under Chapter VII on 30 May 2003 (Ulriksen et al., 2004: 508). On 28 July 2003, the UN Organization Mission in the DRC (MONUC), that had been present since 1999, also received a Chapter VII mandate for Ituri (UN Security Council, 2003). Iturians widely recognized that the international political support as well as the robust military intervention helped to calm the violence. They saw them as having contributed to creating the space in which the encounters between local actors could take place.^18^

The above shows that there was trans-scalarity in ending the violence in Ituri as there was a consistency of purpose and a division of tasks according to each actor’s comparative advantage. Local actors brought communities back together and spread messages of peace while international actors created the space for these initiatives. Thus, a trans-scalar approach enabled the establishment of a negative peace in Ituri. However, while observers underlined the complementarity of efforts in hindsight, this consistency of purpose and division of tasks did not happen as a result of intentional coordination, but came about in a largely unintentional manner. While it thus shows the high potential of a trans-scalar approach, it also indicates that trans-scalarity can be fortuitous when the purpose and comparative advantages of actors are straightforward.

### Lack of trans-scalarity in transitional justice

In contrast to ending violence, there was no trans-scalarity regarding transitional justice in Ituri. While there were many local initiatives, they were not coordinated with a viable national, regional, or international response. According to local accounts, the main purpose of transitional justice in Ituri was reconciliation.^19^ Reconciliation is defined as “a process of relationship building” (Bloomfield, 2006: 28) whereby the relationships should “contain sufficient trust to manage conflicts between and within communities as they arise” (Kelly and Hamber, 2005: 14). For local actors in Ituri, the goal of re-establishing relationships was central. When meeting former enemy communities, they often used rituals to support reconciliation. An example is the *palaver* in which the elderly re-establish harmony between two persons, two families, or two ethnic groups. It can take several days and at its end, the conflict parties engage in a symbolic act, for instance burying their weapons or slaughtering a lamb. Another ritual is the joking relationship. The conflict parties verbalize their aggressions and insult each other, but it is kept at the level of a joke. As Ndrabu Buju (2002) states, “[j]oking provides the catharsis and transfer of emotions or emotional tensions and the expression of aggressiveness so that the social consensus after reconciliation is not compromise” (p. 27).

Local peacebuilders supported these processes in three main ways. First, they invited communities to engage in joint activities. They organized dance, music, and sport events, held community-gatherings (so-called *barzas*), accompanied people to the market or to another village, and encouraged communities to engage in collective work. The organization *Fleuves d*’*Eau Vive qui Coulent aux Autres* (FLEVICA), for instance, organized collective work, such as reforestation, with different ethnic groups. Out of this collective work, they formed local conflict resolution committees. They also assisted people to visit villages where their ethnic group was a minority, which built confidence.^20^

Second, several local peacebuilders aimed at engaging communities with the past. They organized reconciliation workshops or supported victims’ medical and psychological rehabilitation. One example is the organization *À l*’*École de la Paix* (Ecopaix), which focused on the psychological impact of violence on children. They organized meetings with students from different schools to engage in creative activities such as acting, making movies, or composing songs in order to talk about their experiences during the conflict. The children then raised awareness in their families, neighborhoods, and communities through brochures and open days at school where they performed plays, read poems, sang, and danced.^21^

Third, local peacebuilders mediated between ethnic communities and encouraged truth-telling.^22^ As one member of the *Réseau Haki na Amani* recalled,

We were in a village which was a Hema enclave, but during the war it had been occupied by Lendus and the Hemas wanted to return to their village. So we needed to mediate between them. There were several reunions lasting for a year in total and during these reunions, people slowly started to tell each other the truth. They said, “we were on the battle field together and on this day you did this and that and I did this and that.” They saw that the war had not brought anything positive. So they said, “we would like to return, please can you leave our land so that we can come back to our village?” The others replied that, first, they needed to convince the extremists amongst them. Otherwise, there would be more violence. Second, they said that the people living in the village had already built their houses on the ground so they needed at least six months in order to build a new house in another village. Third, they had already started to cultivate their fields in the region and thus, they needed to be able to consume the harvest of these seeds before leaving. So they agreed that after six months, the other group could come back. As such, they came to an agreement by telling each other the truth and they could also speak their minds, which is a promise for a sustainable peace.^23^

From the perspective of local respondents, the above-described initiatives contributed to reconciliation between ethnic communities. Interviewees pointed to the fact that people moved freely again, that the two communities visited each other, ate, drank, and joked together, went to the same markets, schools, and church services, and did sports together. They also noted that there were inter-ethnic marriages and that even if they found themselves in the village of the other community and it was too late to return home, it was safe to spend the night there. Most of the respondents agreed that the relationships had significantly improved since 2003.

Yet, while interviewees acknowledged that local initiatives positively influenced reconciliation, they also pointed to their limits. As one observer said, “rituals are good for disputes between small groups, but how to respond to large-scale massacres?”^24^ They particularly lamented the fact that victims and perpetrators never had the chance to tell each other the truth in an institutionalized setting. This was precisely the area in which international actors had the “most pertinent knowledge, experience and capacity for action” (Millar, 2021: 640), in other words a comparative advantage. Yet, they did not share the same purpose of reconciliation. The dynamics around the Truth and Reconciliation Commission (TRC) most clearly illustrate this.

The TRC was set up in 2003 as foreseen in the “Global and Inclusive Agreement.” It had the mandate to “establish the clear and objective truth on the historical reality of facts, crimes and human rights violations.”^25^ However, it ended up focusing on only two types of activities. First, it alleviated tensions between political actors that had not been addressed in the peace negotiations. Second, it organized seminars on peace and in rare cases mediated in community conflicts. A mechanism for truth-seeking was foreseen in the form of victim hearings on massive human rights violations, but never materialized.^26^ Some individuals wanted to bring cases to the TRC, but it did not have the necessary resources to hear them (Ngoma-Binda, 2008). Thus, it remained focused on short-term conflict resolution, rather than on promoting truth-seeking (Commission Vérité et Réconciliation, 2007; Davis and Hayner, 2009: 22).

This was influenced by the fact that international actors did not consider the TRC as an end in itself, but rather as a means to achieve peaceful elections, which was their main priority during the transition period from 2003 to 2006 (Autesserre, 2010; Hellmüller, 2013). The United Nations Development Programme (UNDP), for instance, had only a small project on the TRC while providing large-scale support to the electoral process (Faubert, 2006: 3–4; Kahorha, 2009). A former member of the commission recalled, “during the transition, the TRC worked on the peaceful cohabitation and pacification of the country because elections were to be conducted.”^27^ Activities, such as truth-seeking, could have put the elections at risk and were thus not prioritized (Kahorha, 2009; Kuye Ndondo, 2004). Therefore, once the elections were held, the activities of the TRC were also suspended. While this was foreseen as such, some TRC members suggested a follow-up in their final report given the TRC’s incomplete achievement of its mandate (Commission Vérité et Réconciliation, 2007). They asked the international community for financial support (USIP, n.d.), but it never materialized.

Respondents in Ituri either did not know of the TRC or perceived it as a failure. As one interviewee stated, “[s]ome Iturian representatives left for Kinshasa, but we never knew what they did there. We expected meetings to tell each other the truth and based on this truth, reconcile. This has never taken place.”^28^ Many observers deeply regretted the absence of an institutionalized truth-seeking mechanism. As one respondent mentioned, “after the war, people were left with so many stories, [. . .] no one has addressed it yet.”^29^ Thus, the TRC is locally perceived as having failed to contribute to reconciliation.

The above shows the absence of trans-scalarity in transitional justice. For local actors, the main purpose of transitional justice was reconciliation, but international actors had different purposes, mostly related to securing the elections. There was thus no adequate division of tasks according to each actor’s comparative advantage. This led to a situation in which local initiatives were not complemented by efforts at broader scales, as the TRC did not contribute the missing pieces in terms of institutionalized truth-seeking to local efforts to promote reconciliation. As one interviewee put it, “Ituri has already started the process of reconciliation by itself [. . .]. But it needs to be cemented. This is the task of the international community.”^30^

This lack of trans-scalarity in transitional justice initiatives contributed to the absence of a positive peace in Ituri in that relationships were not fundamentally transformed. While local respondents acknowledged the improved situation when comparing it to the atrocious violence between 1999 and 2003, a different picture emerged when the reference point was not a negative, but a positive peace. Interviewees underlined the continued circulation of arms seen as an indication of a trust deficit, the lack of complete return of displaced persons to villages of close cohabitation with the other community, and the high divorce rate of inter-ethnic couples. They also recounted that suspicion between ethnic communities was still present and that parents continued to transmit stereotypes about the other ethnic group to their children. Many interviewees also stated that they themselves could not forget what had happened during the war, let alone forgive.

## Conclusion

This article analyzed trans-scalarity in the peace process in Ituri. I argued that given the complex nature of the conflict, a trans-scalar approach is needed. However, while there was a consistency of purpose and a division of tasks according to comparative advantages across different scales for ending the violence, the same was not true for transitional justice where international efforts did not relevantly complement local initiatives because they pursued different objectives. The result was a negative, rather than a positive peace.

What would a trans-scalar approach to transitional justice have changed? First, a consistency of purpose would have enabled local and international actors to jointly pursue an agreed objective. Second, it would have meant to privilege the knowledge of local actors as they are closest to where the implementation of transitional justice programs occurs. In that way, international actors could have become aware of the local priority to promote reconciliation and identify relevant activities to complement locally led initiatives, such as supporting an institutionalized truth-seeking mechanism. While the article does not suggest replacing local efforts with international ones, it argues that they need to complement each other in a more purposeful way, so that they become trans-scalar. This requires, first and foremost, openness by international actors to become aware of local priorities and to consider local actors as true partners in peacebuilding and transitional justice efforts.

The article provided empirical insights on trans-scalarity drawn from the case of Ituri. It further developed the concept’s theoretical foundations by extending the analysis to situations of a negative peace and focusing on a particular aspect of a positive peace, namely transitional justice. It showed that while trans-scalarity may unfold spontaneously regarding negative peace as the objective of ending atrocities is widely shared and the comparative advantages of local and international actors are straightforward, to achieve the much more complex objective of a positive peace, coordination is needed to ensure a consistency of purpose in terms of priorities and to agree on an adequate division of tasks.

As a next step in the debate, it is important to explore more carefully the purposes of different actors and to create a discussion around aligning their objectives with a parity of esteem for all actors involved in the endeavor to build peace. Thereby, the widespread consensus on the need to end violence could be capitalized on to build more coherent approaches also in other areas. This article has focused on transitional justice, but further research could extend the analysis to other aspects of peacebuilding, such as mediation, security, socio-economic aspects, or governance issues. Indeed, these sub-fields are characterized by similar debates around the local turn and the need for novel approaches. In that sense, mainstreaming trans-scalarity in peace research could enable better coordinated and more purposeful peacebuilding efforts across its different sub-fields.

## Supplemental Material

sj-pdf-1-cac-10.1177_00108367211059448 – Supplemental material for A trans-scalar approach to peacebuilding and transitional justice: Insights from the Democratic Republic of Congo Click here for additional data file. Supplemental material, sj-pdf-1-cac-10.1177_00108367211059448 for A trans-scalar approach to peacebuilding and transitional justice: Insights from the Democratic Republic of Congo by Sara Hellmüller in Cooperation and Conflict

## Notes

1. Exceptions include Vinck and Pham (2008), Davis (2013), Tunamsifu (2015), and Arnould (2016).

2. Exceptions include Sriram (2007, 2009a, 2009b) and Sharp (2018).

3. I focus on transitional justice as it is directly concerned with the transformation of societal relationships at the core of establishing a positive peace. Some authors distinguish transitional from transformative justice (Lambourne, 2009, 2014; Lambourne and Rodriguez Carreon, 2016). In that perspective, *transitional* justice focuses on a relatively narrow conception of justice related to civil and political rights in terms of addressing violations of international human rights and humanitarian law, while *transformative* justice takes a long-term view extending the notion of justice to address also economic, cultural, structural, and everyday violence (Gready and Robins, 2014; Sharp, 2019). Despite the difference in focus, however, I argue that both approaches aim at a positive peace as they seek to influence societal relationships beyond ending physical violence.

4. I conducted research for this article in the framework of a project on the interaction between local and international peacebuilders in Ituri.

5. I chose the interview sites according to the location of historic events (e.g. sites of massacres, places of violence outbreaks, heavily polarized locations, etc.), access and security, and ethnic diversity.

6. When I arrived in a village, I presented myself to the local chief and explained the purpose of my visit. In most cases, the local chief then suggested interview partners. The only query I forwarded was gender and age diversity either within or across respondent groups.

7. I also conducted some focus group discussions (FGDs) with staff of local and international peacebuilding organizations.

8. I only transcribed and cited the informal discussions when the respondents consented to them being used in publications.

9. For the sake of clarity in the case study, I define local as the sub-state level, national as the state level, regional as encompassing several states, international as encompassing several regions, and global as encompassing all of the above. Yet, I acknowledge that these spheres and especially what counts as “local” are socially constructed and different geographies overlap.

10. Interviews #11 UN representative, Bunia; #82 district authority, Bunia.

11. Interviews #59 local chief, Ituri; #74 local woman, Desa; #82 district authority, Bunia; #89 local chief, Ituri; #95 UN representative, Bunia; #107 former belligerant, Kinshasa and FGDs #60 population groups, Katoto; #69 population groups, Fataki.

12. Interview #12 UN representative, Bunia.

13. Despite episodic flare-ups of violence after 2003, the conflict between Hema and Lendu ethnic groups has largely remained below the standard threshold of a major war of 1000 battle-related deaths, and therefore, can be characterized as a situation of a negative peace. See Sundberg et al. (2012).

14. UCDP Non-State Conflict Dataset, see Sundberg et al. (2012).

15. FGD #66 with population groups, Jiba.

16. Interview #81 local woman, Kasenyi.

17. Interview #54 local peacebuilder, Bunia.

18. Interviews #6 local peacebuilder, Bunia; #21 local peacebuilder, Mahagi; #31 local chief, Ituri; #91 local peacebuilder, Bunia.

19. Interviews #8 local peacebuilder, Bunia; #43 local peacebuilder, Bunia; #59 local chief, Ituri; #74 local woman, Desa and FGDs #60 population groups, Katoto; #69 population groups, Fataki.

20. Interviews #8 local peacebuilder, Bunia; #43 local peacebuilder, Bunia; #48 local peacebuilder, Bunia; and FGD #1 local peacebuilders, Bunia.

21. Interviews #8 local peacebuilder, Bunia; #43 local peacebuilder, Bunia; #48 local peacebuilder, Bunia.

22. Interviews #6 local peacebuilder, Bunia; #21 local peacebuilder, Mahagi; #54 local peacebuilder, Bunia and informal discussion #120 local peacebuilder, Bunia.

23. Interview #54 local peacebuilder, Bunia.

24. Participant at The United Nations Development Programme (UNDP) workshop on diagnostic of social cohesion in Ituri, 31 May to 2 June 2012.

25. Law 04/018 of 30 July 2004, at: http://www.leganet.cd/Legislation/DroitPenal/Loi01.18.30.07.2004.CVR.htm, consulted 17 July 2021.

26. Article 8 in Law 04/018 of 30 July 2004, at: http://www.leganet.cd/Legislation/DroitPenal/Loi01.18.30.07.2004.CVR.htm, consulted 17 July 2021.

27. Interview #43 local peacebuilder, Bunia.

28. Interview #54 local peacebuilder, Bunia.

29. Interview #43 local peacebuilder, Bunia.

30. Interview #107 former belligerant, Kinshasa.
