# Supplementary material for: A trans-scalar approach to peacebuilding and transitional justice: Insights from the Democratic Republic of Congo
Source: Coop Confl. 2021 Dec 29;57(4):415–32. doi: 10.1177/00108367211059448 (PMC9667079; doi:10.1177/00108367211059448)
Supplement: sj-pdf-1-cac-10.1177_00108367211059448 – Supplemental material for A trans-scalar approach to peacebuilding and transitional justice: Insights from the Democratic Republic of Congo [file sj-pdf-1-cac-10.1177_00108367211059448.pdf]

Online Appendix: "A Trans-Scalar Approach to Peacebuilding and Transitional Justice: Insights from the Democratic Republic of Congo", Sara Hellmüller

---

Interviews

Interview #2 with professor, Bunia, 2011
Interview #4 with local peacebuilding actor, Bunia, 2011
Interview #5 with local peacebuilding actor, Bunia, 2011
Interview #6 with local peacebuilding actor, Bunia, 2011
Interview #8 with local peacebuilding actor, Bunia, 2011
Interview #10 with UN representative (national staff), Bunia, 2011
Interview #11 with UN representative (international staff), Bunia, 2011
Interview #12 with UN representative (national staff), Bunia, 2011
Interview #13 with district authority, Bunia, 2011
Interview #14 with district authority, Bunia, 2011
Interview #15 with local peacebuilding actor, Bunia, 2011
Interview #16 with UN representative (international staff), Bunia, 2011
Interview #17 with local peacebuilding actor, Bunia, 2011
Interview #18 with UN representative (national staff), Bunia, 2011
Interview #19 with local peacebuilding actor, Bunia, 2011
Interview #20 with local peacebuilding actor, Mahagi, 2011
Interview #21 with local peacebuilding actor, Mahagi, 2011
Interview #25 with external expert, email, 2011
Interview #26 with external expert, phone, 2012
Interview #27 with external expert, phone, 2012
Interview #28 with professor, Geneva, 2012
Interview #29 with local chief, Ituri, 2012[1]
Interview #31 with local chief, Ituri, 2012
Interview #34 with local chief, Ituri, 2012
Interview #36 with local peacebuilding actor, Nyankunde, 2012
Interview #37 with district authority, Bunia, 2012

---

[1] I do not specify the villages in which I conducted the interviews with local chiefs as this would allow for their identification.

Interview #38 with local peacebuilding actor, Bunia, 2012

Interview #39 with UN representative (national staff), Bunia, 2012

Interview #41 with representative of judiciary, Bunia, 2012

Interview #42 with professor, Bunia, 2012

Interview #43 with local peacebuilding actor, Bunia, 2012

Interview #44 with UN representative (international staff), Bunia, 2012

Interview #45 with UN representative (international staff), Bunia, 2012

Interview #46 with UN representative (international staff), Bunia, 2012

Interview #47 with UN representative (national staff), Bunia, 2012

Interview #48 with local peacebuilding actor, Bunia, 2012

Interview #49 with district authority, Bunia, 2012

Interview #50 with representative of judiciary, Bunia, 2012

Interview #51 with UN representative (international staff), Bunia, 2012

Interview #52 with international organization representative (national staff), Bunia, 2012

Interview #54 with local peacebuilding actor, Bunia, 2012

Interview #55 with representative of judiciary, Bunia, 2012

Interview #56 with local peacebuilding actor, Bunia, 2012

Interview #57 with local peacebuilding actor, Bunia, 2012

Interview #58 with UN representative (national staff), Bunia, 2012

Interview #59 with local chief, Ituri, 2012

Interview #62 with local chief, Ituri, 2012

Interview #63 with local chief, Ituri, 2012

Interview #65 with local chief, Ituri, 2012

Interview #67 with local chief, Ituri, 2012

Interview #68 with local peacebuilding actor, Kpandroma, 2012

Interview #72 with UN representative (international staff), Bunia, 2012

Interview #73 with international organization representative (national staff), Tchele, 2012

Interview #74 with local woman, Desa, 2012

Interview #75 with local chief, Ituri, 2012

Interview #76 with local chief, Ituri, 2012

Interview #77 with secretary of local chief, Ituri, 2012

Interview #78 with local chief, Ituri, 2012

Interview #79 with local judge, Tchomia, 2012

Interview #80 with secretary of local chief, Ituri, 2012

Interview #81 with local woman, Kasenyi, 2012

Interview #82 with district authority, Bunia, 2012

Interview #83 with local peacebuilding actor, Bunia, 2012

Interview #84 with professor, Bunia, 2012

Interview #85 with local peacebuilding actor, Bunia, 2012

Interview #86 with local peacebuilding actor, Bunia, 2012

Interview #87 with local peacebuilding actor, Bunia, 2012

Interview #88 with local chief, Ituri, 2012

Interview #89 with local chief, Ituri, 2012

Interview #90 with local chief, Ituri, 2012

Interview #91 with local peacebuilding actor, Bunia, 2012

Interview #92 with local peacebuilding actor, Bunia, 2012

Interview #93 with UN representative (national staff), Bunia, 2012

Interview #94 with professor, Bunia, 2012

Interview #95 with UN representative (national staff), Bunia, 2012

Interview #96 with professor, Kinshasa, 2012

Interview #97 with former district authority, Kinshasa, 2012

Interview #98 with UN representative (national staff), Kinshasa, 2012

Interview #99 with national politician, Kinshasa, 2012

Interview #100 with civil society representative, Kinshasa, 2012

Interview #101 with civil society representative, Kinshasa, 2012

Interview #102 with professor, Kinshasa, 2012

Interview #103 with national politician, Kinshasa, 2012

Interview #104 with national politician, Kinshasa, 2012

Interview #105 with UN representative (international staff), Kinshasa, 2012

Interview #106 with national elite actor, Kinshasa, 2012

Interview #107 with former belligerent, Kinshasa, 2012

Interview #108 with national politician, Kinshasa, 2012

Interview #109 with national politician, Kinshasa, 2012

Interview #110 with local peacebuilding actor, Bunia, 2012

Interview #111 with external expert, phone, 2012

Interview #112 with external expert, phone, 2013

Interview #113 with local peacebuilding actor, Bunia, 2013

Interview #115 with local peacebuilding actor, Bunia, 2013

Interview #116 with local peacebuilding actor, Bunia, 2013

Interview #117 with local chief, Ituri, 2013

Interview #118 with local chief, Ituri, 2013

Interview #125 with local peacebuilding actor, Bunia, 2013

Interview #127 with local peacebuilding actor, Bunia, 2013

Interview #128 with international organization representative (national staff), Bunia, 2013

Interview #130 with external expert, Geneva, 2010-2013

FGDs

FGD #1 with local peacebuilding actors, Bunia, 2011

FGD #3 with professor and local peacebuilding actor, Bunia, 2011

FGD #7 with population groups, Bembeyi, 2011

FGD #9 with district authorities, Bunia, 2011

FGD #22 with international organization representatives (national staff), Kinshasa, 2011

FGD #23 with donor representatives, Kinshasa, 2011

FGD #24 with international organization representatives (national staff), Kinshasa, 2011

FGD #30 with population groups, Bogoro, 2012

FGD #32 with population groups, Nyankunde, 2012

FGD #33 with population groups, Nyankunde, 2012

FGD #35 with population groups, Komanda, 2012

FGD #40 with local peacebuilding actors, Bunia, 2012

FGD #53 with UN representatives (international staff), Bunia, 2012

FGD #60 with population groups, Katoto, 2012

FGD #61 with population groups, Djugu, 2012

FGD #64 with population groups, Drodro, 2012

FGD #66 with population groups, Jiba, 2012

FGD #69 with population groups, Fataki, 2012

FGD #70 with population groups, Fataki, 2012

Informal discussions

Informal discussion #71 with professor, Bunia, 2012

Informal discussion #114 with professor, Bunia, 2013

Informal discussion #119 with external expert, Bunia, 2013

Informal discussion #120 with local peacebuilding actor, Bunia, 2013

Informal discussions #121 with local peacebuilding actor, Bunia, 2011-2013

Informal discussions #122 with UN representative (international staff), Bunia, 2012-2013

Informal discussion #123 with UN representative (international staff), Bunia, 2013

Informal discussions #124 with UN representative (international staff), Bunia, 2012-2013

Informal discussion #126 with professor, Bunia, 2013

Informal discussion #129 with UN representative (international staff), Bunia, 2013

Informal discussion #131 with international organization representative (international staff), Bunia, 2013

Informal discussion #132 with international organization representative (national staff), Bunia, 2013

Informal discussion #133 with local peacebuilding actors, Bunia, 2012-2013

Informal discussions #134 with UN representative (international staff), Bunia, 2011-2013

Informal discussions #135 with moto taxi drivers, Ituri, 2011-2013
